# Ascites as First Atypical and Only Clinical Manifestation of De Novo Follicular Lymphoma

**DOI:** 10.3390/medicina58101381

**Published:** 2022-10-01

**Authors:** Natasa D. Zdravkovic, Vesna M. Grbovic, Radisa H. Vojinovic, Radojica V. Stolic, Jelena Z. Zivic, Mladen M. Maksic, Ziva P. Zivic, Nebojsa V. Andjelkovic, Milos Z. Zivic, Zeljko D. Todorovic

**Affiliations:** 1 Faculty of Medical Sciences, Department of Internal Medicine, University of Kragujevac, 34000 Kragujevac, Serbia; 2 Clinic of Gastroenterohepatology, University Clinical Center, 34000 Kragujevac, Serbia; 3 Faculty of Medical Sciences, Department of Physical Medicine and Rehabilitation, University of Kragujevac, 34000 Kragujevac, Serbia; 4 Clinic of Physical Medicine and Rehabilitation, University Clinical Center, 34000 Kragujevac, Serbia; 5 Faculty of Medical Sciences, Department of Radiology, University of Kragujevac, 34000 Kragujevac, Serbia; 6 Clinic of Radiology, University Clinical Center, 34000 Kragujevac, Serbia; 7 Clinic of Nephrology, University Clinical Center, 34000 Kragujevac, Serbia; 8 Clinic of Hematology, University Clinical Center, 34000 Kragujevac, Serbia; 9 Faculty of Medical Sciences, Department of Dentistry, University of Kragujevac, 34000 Kragujevac, Serbia

**Keywords:** follicular lymphoma, ascites, gastrointestinal lymphoma

## Abstract

Follicular lymphoma is the most common indolent non-Hodgkin’s lymphoma and is usually initially detected in lymph nodes. Primary extranodal NHL is most commonly primarily localized in the gastrointestinal tract. We present one unusual case of ileum FL with ascites as the first clinical sign. The 73-year-old female patient was presented to the emergency department for evaluation of mild abdominal pain and abdominal swelling that had been going on for three days followed by bloating and occasional pain in the spine. The abdominal contrast-enhanced CT revealed the contrast stagnation in the distal part of the ileum. The ileum wall about 11 cm in length was thickened up to 2.9 cm and the tumor mass infiltrated all layers of ileum mesenteric lymphadenopathy up to 2 cm in diameter and significant ascites. On the upper ileum wall, the vegetative mass was described 3 cm in diameter. The patient had an emergent laparotomy with the ileocolic resection and latero-lateral ileocolic anastomosis. The microscopy finding of terminal ileum and the regional lymph nodes showed domination of cleaved cells with irregular nuclei which correspond to centrocytes. There were 0–15 large non-cleaved cells corresponding to centroblast in the microscopy high-power field. The final diagnosis was follicular lymphoma, the clinical stage 2E and histological grade by Berard and Mann criteria 1–2.

## 1. Introduction

Follicular lymphoma (FL) is the most common indolent non-Hodgkin’s lymphoma (NHL) in the Western world and is usually initially detected in lymph nodes [1]. Extranodal occurrence most often happens as a result of disseminated nodal disease but the extranodal site can also be primarily localized. Primary extranodal NHL is most commonly primarily localized in the gastrointestinal tract, accounting for 30–40% of all primary extranodal NHL [2]. The most common histological subtypes of primary gastrointestinal NHL are mucosal-associated lymphoid tissue (MALT) lymphoma and diffuse large B cell lymphoma (DLBCL) [3]. Follicular lymphoma accounts for only 1–3.6% of gastrointestinal NHL [3]. Based on the literature, duodenum is the most frequent site of primary gastrointestinal FL (PG-FL) followed by jejunum and ileum [4]. Yamamoto et al. reported that most cases of follicular lymphoma are asymptomatic. The abdominal pain is the most common symptom and 28.7% of the examined patients had it. The symptoms of intestinal obstruction, such as nausea and vomiting, were present in 8.0% and the symptoms of intestinal bleeding, such as tarry stool or hematochezia, were present in 6.0%. In patients with small-intestinal lesions, the intestinal obstruction was more often, whereas bleeding was observed mostly in patients with colorectal lesions [5]. Herein, we present one unusual case of ileum FL with ascites as the first clinical sign.

## 2. Case Presentation

The 73-year-old female patient was presented to the emergency department for evaluation of mild abdominal pain and abdominal swelling that had been going on for three days followed by bloating and occasional pain in the spine. Physical examination of the patient revealed normal vital signs, distended and painless abdomen on deep palpation without rebound tenderness, guarding or palpable masses. Her initial labs showed normal electrolytes with a normal coagulation profile, presence of normocytic anemia, elevated d-dimer 4420 ng/mL, elevated markers of inflammation (C reactive protein 62.9 mg/L, leukocytes 11.03 × 10^9^/L, with a predominance of granulocyte). The serum levels of aspartate and alanine aminotransferase, gamma glutamyl transferase, albumin, amylase and lipase were normal (0–160 U/L). The level of lactate dehydrogenase was high, 410 U/L. Her medical history revealed that she had myocardial infarction two years ago and that she suffers from cardiomyopathy, anemia, hypertension, diabetes and stage 2 chronic kidney disease.

**Figure 1:**
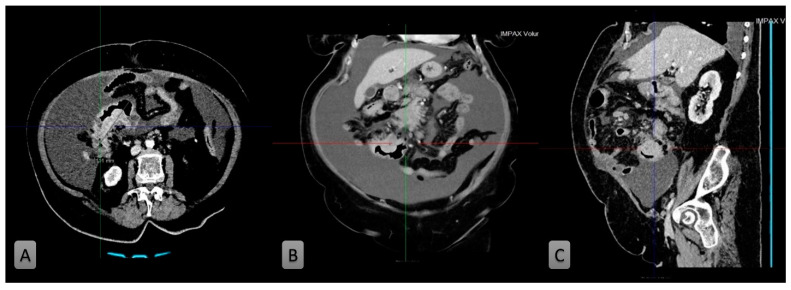
The tumor mass infiltrated all layers of the ileum wall (**A**). The vegetative mass about 3 cm in dimeter on the upper ileum wall (**B**). On all three images, ascites is present (**C**).

After the abdominal ultrasound had showed ascites, computed tomography (CT) was performed. The abdominal contrast-enhanced CT revealed the contrast stagnation in the distal part of the ileum where the ileum wall about 11 cm in length was thickened up to 2.9 cm and the tumor mass infiltrated all layers of the ileum (Figure 1A). On the upper ileum wall, the vegetative mass was described 3 cm in diameter (Figure 1B). Also, CT showed mesenteric lymphadenopathy up to 2 cm in diameter and significant ascites (Figure 1C). Chest and neck CT did not show enlarged lymph nodes.

Ascites is evacuated and cytological analysis of ascites showed the presence of atypical lymphocytes.

**Figure 2:**
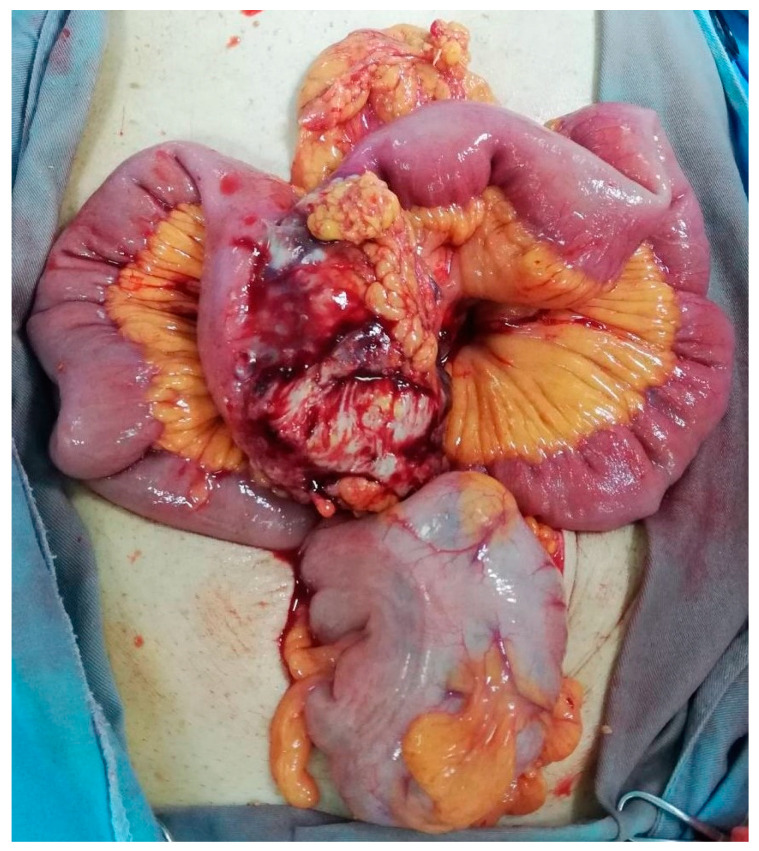
Macroscopic finding.

**Figure 3:**
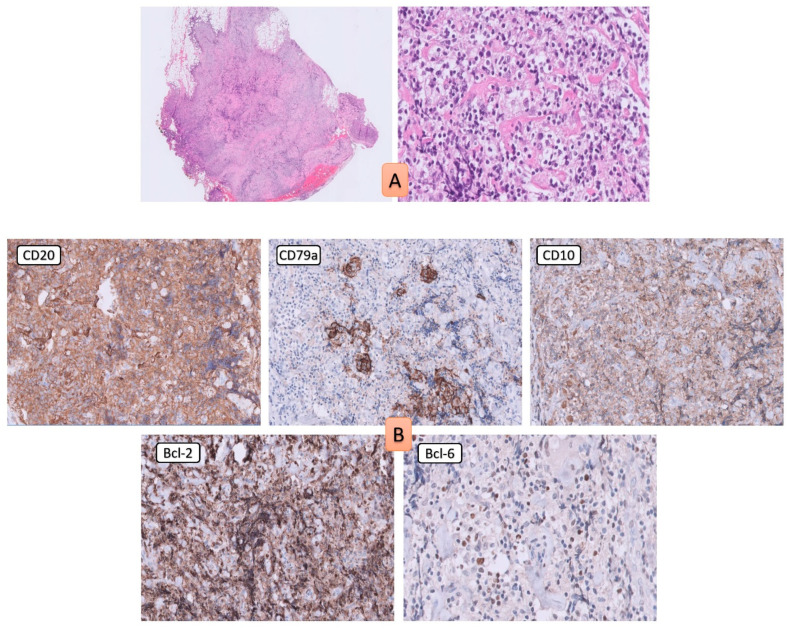
Resected ileum tissue showed centrocyte and centroblast (**A**). Cells were positive on CD20, CD79a, CD10, bcl-2, bcl-6 (**B**).

The patient had an emergent laparotomy. Macroscopic finding showed ileum infilration with vegetative mass (Figure 2). Ileocolic resection and latero-lateral ileocolic anastomosis was done. The microscopy findings of the terminal ileum and the regional lymph nodes showed domination of cleaved cells with irregular nuclei which correspond to centrocytes. In the microscopy high-power field, there were 0–15 large non-cleaved cells corresponding to centroblast (Figure 3A). Immunohistochemically stains showed that those cells were CD20 positive, CD79A positive, CD10 positive, PAX-5 positive, BCL-2 positive, and BCL-6 positive (Figure 3B).

Considering that the bone marrow biopsy did not show lymphoma involvement, the final diagnosis of follicular lymphoma, the clinical stage 2E and histological grade by Berard and Mann criteria 1–2 were made and the patient was started with G-CVP regimen (cyclophosphamide, vincristine, and prednisone plus Gazyva).

## 3. Discussion and Conclusions

Most cases of gastrointestinal FL are classified using the Ann Arbor staging system or the Lugano staging classification of gastrointestinal lymphoma, which is a modified version of the Ann Arbor staging system for gastrointestinal NHL [6]. Unlike nodal FL, where patients most often have a disseminated disease (Ann Arbor stage III and IV), PG-FL is a localized disease with majority of patients in the stage I (Ann Arbor and Lugano) [7]. A higher ratio of localized PG-FL may be due to the definition of PG-FL which excludes FL with both intestinal and massive extraintestinal lesions or due to PG-FL cells originating from B cells in the intestinal mucosae with different mucosal homing receptors. Our patient was classified as the stage IIE by the Ann Arbor and Lugano classification due to the involvement of local mesenteric lymph nodes and infiltration of all intestinal layers including serosa [6].

Follicular lymphoma cells are composed of small to medium-sized cleaved cells (centrocytes) and large non-cleaved cells (centroblasts) [4]. By Berard and Mann criteria, FLs are classified in three histological grades according to the number of centroblasts. More centroblasts mean higher histological grade and the worst prognosis [8]. Yamamoto et al. showed that frequencies of the grade 1, grade 2, and grade 3 in PG-FL are 84.4%, 11.3%, and 4.3%, respectively, while those of the grade 1, grade 2, and grade 3 in nodal FL are 40–60%, 25–35%, and approximately 20%, respectively [5]. Those findings may explain why PG-FL is, in most cases, a localized disease. In correspondence with those findings, our patient had FL histological grade 1–2.

Using Southern blotting, the polymerase chain reaction (PCR) and fluorescence in situ hybridization (FISH), t (14;18) (q32; q21) were found in approximately 60–70% of cases of PG-FL, similar to nodal FL. This translocation causes bcl-2, an anti-apoptotic protein, to be overexpressed, which is thought to be one of the causes of lymphomagenesis [6]. Except on bcl-2, like nodal FL, PG-FL cells are positive on bcl-6, CD10 and pan-B-cells antigens CD19, CD20, CD22, PAX5, CD79a. Immunohistochemical staining for CD10 and bcl-2 is useful for distinguishing FL from mantle lymphoma, lymphoid hyperplasia or MALT lymphoma [9]. In approximately 90% of FL, both CD10 and bcl-2 are expressed. Normal germinal center cells express CD10 but not bcl-2 which distinguishes FL from lymphoid hyperplasia. MALT lymphoma is negative for CD10 and occasionally positive for bcl-2, while mantle cell lymphoma expresses neither CD10 nor bcl-2 [9]. Our patient had a typical FL phenotype with both positive bcl-2 and bcl-6. Studies have shown that FL histological grades 1–2 are bcl-2 negative in 10–20% of cases, the rate of negativity is about 50% in the grade 3. This is in correspondence with the findings that bcl-2-/bcl-6+ FL are usually in higher grade than bcl-2+/bcl-6+ FL [10].

For PG-FL, four treatments are used: watch and wait strategy, radiation therapy, rituximab monotherapy and immunochemotherapy [10]. Considering that radiation of the small bowel is associated with chronic radiation enteritis and disease burden in a form of ascites, we decided to perform the immunochemotherapy treatment with G-CVP regimen. The patient received the first cycle of therapy.

## Data Availability

All data can be found in University Clinical Center, 34000 Kragujevac, Serbia.
